# The Strengths and Difficulties Questionnaire (SDQ): Factor Structure and Gender Equivalence in Norwegian Adolescents

**DOI:** 10.1371/journal.pone.0152202

**Published:** 2016-05-03

**Authors:** Tormod Bøe, Mari Hysing, Jens Christoffer Skogen, Kyrre Breivik

**Affiliations:** 1 Regional centre for child and youth mental health and child welfare, Uni Research Health, Bergen, Norway; 2 Division of Mental Health, Department of Public Mental Health, Norwegian Institute of Public Health, Bergen, Norway; 3 Alcohol and Drug Research Western Norway, Stavanger University Hospital, Stavanger, Norway; University of Geneva, SWITZERLAND

## Abstract

Although frequently used with older adolescents, few studies of the factor structure, internal consistency and gender equivalence of the SDQ exists for this age group, with inconsistent findings. In the present study, confirmatory factor analysis (CFA) was used to evaluate the five-factor structure of the SDQ in a population sample of 10,254 16–18 year-olds from the youth@hordaland study. Measurement invariance across gender was assessed using multigroup CFA. A modestly modified five-factor solution fitted the data acceptably, accounting for one cross loading and some local dependencies. Importantly, partial measurement non-invariance was identified, with differential item functioning in eight items, and higher correlations between emotional and conduct problems for boys compared to girls. Implications for use clinically and in research are discussed.

## Introduction

Epidemiological studies have demonstrated that 13–25% of adolescents will meet the criteria for a mental disorder during their lifetime [1, 2]. Adolescence is an important time point for assessment, and possible early intervention as many mental health problems commonly emerge during this age period [3]. Adolescent mental health problems often go unnoticed [4], and use of screening instruments can aid early detection of these problems and may facilitate early intervention and access to effective treatments.

The Strengths and Difficulties Questionnaire (SDQ) [5, 6] is a brief assessment questionnaire for mental health problems that was originally developed for children between 11–16, but has recently been used also in older age groups. It covers a broad range of mental health symptoms including conduct problems, hyperactivity-inattention, emotional symptoms, and peer problems, as well as prosocial behaviours. Multiple informants can complete the SDQ, including parent, teacher and self-report, and information about both symptom and impact scores are included.

Previous psychometric evaluations of the self-report version have given somewhat mixed results. The scale has shown good concurrent and discriminant validity as it correlates strongly with related scales such as the Youth Self-Report and the Revised Children’s Manifest Anxiety Scale [7, 8] and seems able to discriminate adequately between normative and clinical populations. As for the parent and teacher version of the SDQ, previous research has located potential problems with the internal structure of the test. First, Goodman’s theoretically derived five-factor structure has only received inconsistent support when tested by the use of confirmatory factor analysis (CFA) [9–11]. Anna Goodman and her colleagues [12] argue that the SDQ does not seem to have “a very clean internal factor structure but that the hypothesized five subscales may nonetheless provide a passable description” ([12], p 1188). In support of this view some researchers have found that the fit of the five-factor structure has become acceptable after some modest modifications on the model [13–15]. In a study using data from more than 3000 participants from five European countries, Ortuño-Sierra et al. [16] found acceptable fit for a modified five-factor model for the total sample, and for all individual countries bar one (Ireland). The study also found partial invariance for many items across countries, suggesting that various country-specific modifications may be required for appropriate screening.

Second, even in studies that have supported the five-factor structure, the internal reliability of the subscales has often been found to be poor (e.g. Chronbach’s *α*s <0.70, see Stone et. al. [17]). Stone et al. [18] have, however, argued convincingly that the poor internal reliability found in previous research might simply be a consequence of assessing it by the use of Chronbach’s *α* which they point out have been criticized by many contemporary statisticians (e.g., [19]). The poor internal reliability found in previous studies might moreover be a consequence of typically treating the items as continuous rather than ordinal which might both lower the internal reliability [20] and lead to both to an improper factor structure and poorer model fit [21]. In support of this, van de Looij-Jansen, Goedhart and de Wilde [22] found that the internal reliability of all sub scales bare two (Conduct and Peer problems) became acceptable when using ordinal *α* instead of Chronbach´s *α*. Using ordinal *α*, acceptable reliability was also found in a study of Spanish adolescents [14], and for the total sample in a study comparing the SDQ across five European countries, although the strength of the reliability coefficients varied by subscale and country [16]

In the present study we aimed at evaluating the proposed five-factor structure and the internal consistency of the self-report version of the SDQ in a recent large population sample of 16–18 year-olds in Norway. These youths are somewhat older than the intended age group for the test (11–17 year olds; see http://www.sdqinfo.org/).

Few previous investigations have reported psychometric results on the self-reported SDQ for older adolescents, and these have given inconsistent results. Yao et al.’s [23] findings indicate that the self-report version of the SDQ might not be suitable for older adolescents as they found a less than satisfactory fit of the five-factor model in their Chinese sample of 15–18-year-olds, while the fit was excellent among 11–14-year-olds. One study from Norway using data collected in 2002, found satisfactory fit for the five-factor structure among 16–19-year-olds, but noted that accounting for some correlated error terms improved model fit significantly [9]. Ortuño-Sierra et al. [14] found acceptable fit for a five-factor solution with correlated error terms in a study of a Spanish population sample of 11–19 years olds. Their results also suggested that the fit of the model was measurement invariant across age, i.e. that the fit of the model was comparable for younger (11–15 years) and older adolescents (16–19 years). In a different investigation, Ortuño-Sierra et al. [15] also found acceptable model fit for a five-factor solution using a modified version of the SDQ with five response options (compared to three in the original version) after accounting for correlating error terms. The study found strong measurement invariance by age, suggesting that the fit of the SDQ was not poorer for older (17–18 years) compared to younger (14–16 years) participants, although the fit of both models were questionable (i.e. both models has CFI < .90 and TLI < .90). It also appears this analysis did not account for the ordinal categorical structure of the response options. The limited number of studies and inconsistent results warrants further replication and investigation of the five-factor structure of the self-report SDQ in samples of older adolescents.

In the present study we also aimed to explore whether the SDQ is measurement invariant across gender in our sample. The study of measurement invariance is highly important as non-invariant measures might lead to biased group mean comparisons and inadequate selection decisions (e.g. screening) about individuals [24]. Surprisingly few studies have examined whether the self-report version is measurement invariant across gender. Also in this area, there are inconsistent findings. Some studies, one on Norwegian 11–16-year-olds [25] and one on Spanish adolescents [14] concluded that the scale was measurement invariant across gender, whereas another study found partial measurement invariance for gender using a modified version of the SDQ where response options had been changed from three to five [15]. In the study by Rønning et al. [25] a chi-square difference test was used to compare factorial invariance between boys and girls and it is uncertain whether this procedure represents a robust test of measurement invariance [26]. In the studies by Ortuño-Sierra et al [14, 15] the between-models difference in CFI was used to judge measurement invariance. This procedure is validated in several simulation studies, but with varying recommendations regarding which cut-off to use to determine invariance [27–29], and little is known about the implications of using a certain different cut-off. In, one study from the Netherlands using a sample of 11–16-year-olds found some indications of strong factorial *non*-invariance for gender, but still concluded that there was a reasonable degree of congruence between boys and girls and that valid comparisons between genders could be made [22]. One way of supporting their conclusion would have been to investigate the practical implications (such as whether the non-invariance was reflected in mean differences between genders) of this measurement non-invariance.

In summary, the few studies, discrepant findings and the frequent use of the SDQ for adolescents older than 16 years suggest there is a need for further studies examining the psychometric properties of the SDQ in this age group. Furthermore, there is a need for explicitly testing whether the SDQ is measurement invariant across gender and to investigate any practical implications of non-invariance. The aims of the current study were thus to investigate the factor structure of the SDQ in sample of Norwegian 16–18-year-olds, to assess measurement invariance for gender using a multigroup CFA framework and lastly, to investigate differential item functioning in the SDQ for gender.

## Materials and Methods

In this population-based study, we employed information from the youth@hordaland survey of adolescents in the county of Hordaland in western Norway. All adolescents born between 1993 and 1995 and all students attending secondary education during spring 2012 were invited to participate. The main aim of the survey was to assess mental health problems and health service use in adolescents, with a special emphasis on the prevalence of mental health problems. The data were collected during spring 2012. Adolescents in upper secondary education received information via e-mail, and one school hour was allotted for them to complete the questionnaire at school. Those not in school received information by postal mail to their home addresses. The questionnaire was web-based, and covered a broad range of mental health issues, daily life functioning, use of health care and social services, and demographic background variables. Uni Research Health collaborated with Hordaland County Council in conducting the study. The study was approved by the Regional Committee for Medical and Health Research Ethics (REC) in Western Norway. In accordance with the regulations from the REC and Norwegian health authorities, adolescents aged 16 years and older can make decisions regarding their own health, and may thus give consent themselves to participate in health studies. Parents/guardians have the right to be informed, and in the current study, all parents/guardians received written information about the study in advance. If the adolescents decided to participate they indicated if they wanted to participate in the study as a whole, or they could choose three options to specify their level of consent: 1) complete the questionnaire, which meant to fill out the online questionnaire from which the data from the current study originated. The two other options were: 2) “obtain information from parent questionnaire” meaning that the adolescent responses could be linked to information that was obtained from their parents who completed a similar questionnaire (later administered to the parents of those adolescents who consented). The third option “linking data to national registries” indicated consent for linking the information in the questionnaire to administrative registers (of for example school performance and family income). The current study used adolescent questionnaire data only.

### Sample

From a target population of 19,430, 10,254 participated yielding a participation rate of 53%. About half of the sample was male (47.3%) and the mean age was 17 years. Most participants were high school students (97.7%), some undertook vocational training (1.5%) and a few reported not being in school (0.8%). The majority of parents had college/university degrees (45.6%) or secondary education (43.8%), and 10.5% had primary school as the highest level of completed education.

### SDQ measurements

In the youth@hordaland study, adolescents completed the self-report version of the SDQ [5, 6]. The SDQ is available from http://www.sdqinfo.org and can be downloaded freely. It consists of five subscales, each containing five items. The scales measure emotional symptoms, conduct problems, hyperactivity-inattention, peer relationship problems, and prosocial behaviours. Respondents indicated on a three-point Likert-type scale to which extent a symptom applied to them, using the options “Not true”, “Somewhat true”, or “Certainly true”. Each of the subscales consists of five items, and scale scores range from 0–10. A higher score is indicative of more problems for all subscales, except for the prosocial scale, where higher scores correspond to fewer difficulties in prosocial behaviour.

The internal consistency coefficient Chronbach’s α, was adequate for the SDQ Total difficulties scale (*α* = 0.78) and for the subscale emotional problems (*α* = 0.73), but low for hyperactivity-inattention (*α* = 0.69) and prosocial behaviours (*α* = 0.63), and poor for peer problems (*α* = 0.57), and for conduct problems (*α* = 0.47).

**Fig 1:**
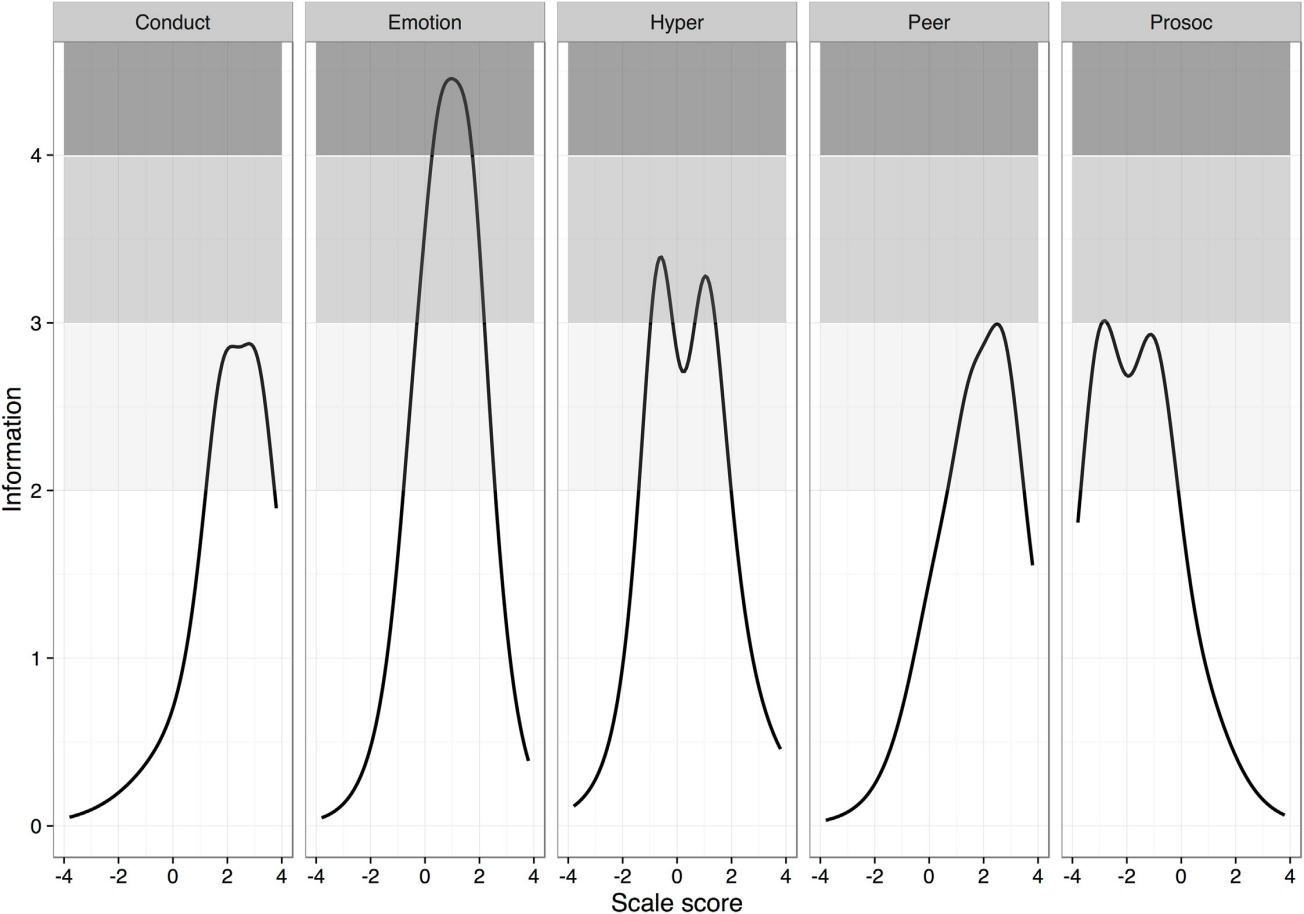
Precision of the subscales of the SDQ along the trait level. Shaded areas correspond to Cronbach’s alphas of 0.5–0.67 (light grey), 0.68–0.75 (medium grey), and 0.76–0.80 (dark grey).

Analyses using ordinal Cronbach’s α, a polychoric correlation-based version of the reliability coefficient [30], however, suggested satisfactory internal consistency for the SDQ Total difficulties scale (*α* = 0.86) and for all subscales (*α*s emotional problems = 0.82, conduct problems = 0.71, hyperactivity-inattention = 0.76, peer problems = 0.75, and prosocial behaviors = 0.77). Reliability was further investigated by item response analyses by fitting a graded response model to each subscale of the SDQ using the R package “ltm” [31]. The precision of the instrument across different trait levels is illustrated in Fig 1.

### Statistical methods

All analyses were conducted using R for Mac version 3.0.1 [32] using libraries Psych (version 1.3.2; [33]) for descriptive and scale-reliability analyses and Lavaan (version 0.5–14; [34]) for confirmatory factor analyses (CFA). The robust-weighted least square (WLMSV) estimator was used in the CFAs due to the highly skewed categorical data (ordinal data with three options). WLMSV uses polychoric correlations for estimations and seems to be reasonably robust to violations of normality [35, 36]. There was less than 2 percent missing on each of the SDQ items. Missing data was handled by pairwise deletion, resulting in only one case being excluded from the analyses. Model fit were assessed using the comparative fit index (CFI) and the root mean square error of approximation (RMSEA). There are several guidelines for interpreting model fit. In the current study, CFI values greater than 0.90 together with RMSEA values of less than 0.08 were considered acceptable [35], whereas CFI values above 0.95 and RMSEA below 0.06 were preferred [37]. We did not rely on the Chi-square statistic as evidence of model fit, as it is highly sensitive to sample size and violations of normality [28, 29].

In order to test for measurement invariance in factor loadings and thresholds across gender (i.e. *strong invariance*), we followed the procedure recommended by Muthén and Muthén [38] for use with delta parameterization. In this approach, the fit of the model of which the loadings and thresholds were held equal between the genders was compared to a (configural invariance) model where the loadings and thresholds (except for the identification items) were free to vary. The model was assumed as being scalar and metric measurement invariant if the decrease in CFI (i.e. ΔCFI) was less than 0.002, as recommended by Meade, Johnson and Braddy [27]. The advantages of using ΔCFI to determine measurement invariance has been confirmed in several simulation studies [28, 29], and Oliden [39] recently demonstrated the utility of the ΔCFI criterion for ordered categorical data as well. If ΔCFI was larger than 0.002, potentially problematic items (based on modification indices [MI]) were inspected and the parameter with the largest MI for factor loading and threshold was freed in tandem, and the model re-run. This iterative process of fitting the model, and re-specifying the model according to the largest modification index was repeated until a model was obtained where the ΔCFI between the model currently being tested and the configural model was less than 0.002.

Finally, we constrained the co-variance of the latent factors to be equal in order to test the model for structural equivalence. Using a similar procedure as described above, we freed parameters with the largest MI for co-variance between the latent factors until a model with a sufficiently good fit was attained.

## Results

The rate of endorsement of the SDQ items can be seen in Table 1.

**Table 1:** Endorsement rates for response categories on each item in the Strengths and Difficulties Questionnaire for the full sample, and separately for boys and girls.

	Not true %			Somewhat true %			Certainly true %		
	Full sample	Boys	Girls	Full sample	Boys	Girls	Full sample	Boys	Girls
SDQ scales									
Emotion									
Somatic	53.88	69.11	40.33	31.3	23.63	38.12	14.82	7.26	21.55
Worries	40.21	54.22	27.77	40.54	34.97	45.50	19.25	10.81	26.72
Unhappy	64.97	81.28	50.47	26.26	14.92	36.33	8.78	3.80	13.20
Clingy	33.01	41.93	25.10	47.62	45.39	49.59	19.37	12.69	25.31
Afraid	73.09	86.78	60.91	20.96	11.04	29.77	5.96	2.17	9.32
Conduct									
Tantrum	53.88	59.74	49.01	36.84	33.21	40.08	9.09	7.05	10.92
Obeys^a^	3.40	4.07	2.81	48.34	50.91	46.07	48.25	45.02	51.11
Fights	92.63	89.96	94.99	6.36	8.80	4.20	1.01	1.24	0.81
Lies	87.12	82.12	91.56	10.30	14.19	6.85	2.58	3.69	1.59
Steals	90.46	88.22	92.44	7.70	9.67	5.94	1.85	2.11	1.61
Hyper									
Restless	32.47	35.07	30.17	50.83	47.51	53.77	16.70	17.42	16.06
Fidgety	47.4	48.59	46.33	42.2	41.28	43.03	10.40	10.14	10.63
Distractible	30.84	34.26	27.80	47.63	47.95	47.33	21.54	17.78	24.87
Reflective^a^	5.94	6.49	5.46	59.76	56.30	62.84	34.30	37.21	31.70
Attends^a^	14.26	13.24	15.17	57.79	57.88	57.71	27.95	28.89	27.13
Peer									
Loner	49.68	47.31	51.79	39.49	41.30	37.87	10.84	11.39	10.34
Friend^a^	2.20	2.30	2.12	10.51	11.08	10.01	87.28	86.62	87.87
Popular^a^	3.58	4.16	3.06	37.77	37.94	37.63	58.65	57.90	59.31
Bullied	91.74	90.88	92.50	6.87	7.66	6.17	1.39	1.46	1.33
Oldbest	59.76	59.63	59.87	32.48	33.14	31.90	7.76	7.22	8.23
Prosocial									
Considerate	1.15	1.71	0.66	16.12	22.61	10.36	82.73	75.68	88.98
Shares	4.56	5.85	3.41	40.45	43.56	37.70	54.99	50.59	58.89
Caring	2.93	4.73	1.33	29.47	42.77	17.67	67.60	52.50	81.00
Kind	3.22	3.84	2.66	24.62	29.31	20.46	72.17	66.85	76.88
Helps out	10.11	12.41	8.08	62.02	62.51	61.57	27.87	25.08	30.35

^a^Positively worded items.

There was a tendency for girls to more often endorse items measuring symptoms of emotional problems and prosocial behaviors. There did not appear to be any gender-particular differences in the response pattern on the other items.

The fit of the five-factor model was not optimal, CFI = 0.898, TLI = 0.085, RMSEA = 0.066, 95% confidence interval of the RMSEA (CI) = [0.065, 0.067]. Inspection of the modification indices suggested that it was necessary to account for a cross-loading between the item *tantrum* from the conduct problems scale and the emotional problems latent factor (*β* = 0.353), and to correlate the error terms of the two items measuring hyperactivity (*restless* and *fidgety*, *r* = .555) and the error terms of the two items measuring inattention (*distract* and *attends*, *r* = .332) in the proposed latent hyperactivity-inattention factor. After these adjustments, an acceptable fit was found for the modified five-factor model to the self-reported SDQ data, CFI = 0.929, TLI = 0.919, RMSEA = 0.055, CI = [0.054, 0.056]. The model was then specified within a framework of a multigroup CFA to test for configural measurement invariance between the genders. The fit of the multi-group model was acceptable (CFI = 0.921, TLI = 0.910, RMSEA = 0.058, CI = [0.057, 0.059]), suggesting that the factor structure and the loading pattern are similar across both genders.

Testing for strong measurement invariance (i.e. constraining the loadings and thresholds) reduced the model fit, CFI = 0.915, TLI = 0.910, RMSEA = 0.057, CI = [0.056, 0.058] compared to the multi-group model. The reduction in CFI between this model and the multi-group model (ΔCFI = 0.006) exceeded the cut-off of 0.002, suggesting that the model was non-invariant. In order to obtain a model with a fit not exceeding these criteria, eight items with large modification items for loading and threshold had to be freed iteratively. The resulting model had an acceptable fit (CFI = 0.920, TLI = 0.912, RMSEA = 0.057, CI = [0.056, 0.058]). The standardized factor loadings and thresholds based on confirmatory factor analysis for the SDQ can be found in Table 2. The eight items with differential item functioning (DIF) are indicated with bold typeface. Note that the remaining *standardized* factor loadings and thresholds might differ slightly between the genders, as it is the *unstandardized* factor loadings and thresholds that have been constrained to be equal.

**Table 2:** Standardized factor loadings and thresholds based on confirmatory factor analysis for the SDQ five-factor model (n = 10,253).

	Girls			Boys		
	Factor loading	Threshold1	Threshold 2	Factor loading	Threshold 1	Threshold2
Emotion						
Somatic	0.578	-0.205	0.783	0.559	-0.210	0.804
Worries	0.670	-0.610	0.607	0.589	-0.569	0.566
Unhappy^a^	0.814	0.012	1.117	0.822	-0.085	0.801*
Clingy^a^	0.595	-0.671	0.665	0.560	-0.867	0.478
Afraid^a^	0.650	0.277	1.321	0.748	0.230	1.133
Conduct						
Tantrum^a^	0.412	-0.025	1.231	0.359	0.364*	1.590*
*Obeys*^a^^b^	0.571	0.028	1.909	0.552	0.538*	2.406*
Fights	0.622	1.647	2.401	0.696	2.114	3.083
Lies	0.621	1.403	2.141	0.650	1.685	2.572
Steals	0.471	1.418	2.089	0.532	1.838	2.708
Hyper						
Restless	0.517	-0.519	0.976	0.439	-0.466	0.875
Fidgety	0.496	-0.107	1.236	0.456	-0.104	1.201
Distractible	0.755	-0.559	0.719	0.731	-0.572	0.736
*Reflective*^a^^b^	0.468	-0.476	1.602	0.499	-0.419	1.422
*Attends*^b^	0.710	-0.643	1.010	0.674	-0.645	1.013
Peer						
Loner	0.573	0.022	1.305	0.499	0.021	1.217
*Friend*^b^	0.745	1.149	1.998	0.737	1.217	2.117
*Popular*^b^	0.706	0.255	1.821	0.673	0.260	1.859
Bullied^a^	0.628	1.439	2.217	0.775*	1.427	2.275
Oldbest	0.422	0.264	1.406	0.418	0.280	1.491
Prosocial						
Considerate	0.806	-2.611	-1.178	0.792	-2.374	-1.071
Shares	0.475	-1.794	-0.228	0.518	-1.810	-0.230
Caring^a^	0.607	-2.217	-0.878	0.635	-1.940*	-0.331*
Kind	0.650	-1.993	-0.731	0.710	-2.017	-0.740
Helpout	0.479	-1.402	0.495	0.504	-1.366	0.483

^a^Items have differential functioning for gender.

^b^Items are positively worded.

* The difference between the parameter value for boys and girls exceed 0.25, corresponding to a small effect size

The magnitude of the standardized factor loadings ranged from 0.412 to 0.814. The results demonstrated that girls had lower thresholds for endorsing the items *caring*, *tantrum* and *obeys*, while boys has a lower threshold for responding “certainly true” to the item *unhappy*. For the items *tantrum*, *obeys* and *caring*, we found larger effect sizes for the differences in parameter estimates, and they appeared for both thresholds. For two other items (*afraid* and *unhappy*) the effect sizes of the differences in parameter estimates were small and mainly apparent in the second threshold. Other threshold differences were very small. One loading was flagged measurement non-invariant as the item *bullied* was somewhat more discriminating between adolescents higher and lower on the peer problems trait (higher factor loading) for boys compared to girls.

In order to convert the parameter estimates into Cohen’s *d*’s, we used the formula provided by Choi, Fan, and Hancock [40] where *K*_2_ represent the estimate for boys, ϕ_1_ and ϕ_2_ is the sample construct variances for girls and boys respectively, *n*_1_ is *n* for girls (5,399), and *n*_2_ is *n* for boys (4,854): $\hat{d}=\frac{\left|{\hat{\kappa }}_{\text{2}}\right|}{\sqrt{(\frac{{\text{n}}_{\text{1}}}{{\text{n}}_{\text{1}}\text{+}{\text{n}}_{\text{2}}}){\hat{\phi }}_{\text{1}}\text{+}(\frac{{\text{n}}_{\text{2}}}{{\text{n}}_{\text{1}}\text{+}{\text{n}}_{\text{2}}}){\hat{\phi }}_{\text{2}}}}$

In order to assess the impact of the items that functioned differently on genders, the difference in latent means between boys and girls in the model taking DIF into consideration was compared to the model where DIF was disregarded (see Table 3). The latent mean estimate was converted to effect size estimates (Cohen’s *d*’*s*) using the formula provided by Choi, Fan, and Hancock ([40]; formula 3, p. 389) for use when one population is used as reference and the latent mean is constrained to zero for that population. It was found that boys had lower latent means than girls on emotional problems, hyperactivity-/inattention and prosocial behavior, whereas boys had greater latent means on conduct problems and peer problems. In the model where DIF was disregarded, the effect sizes of the difference in latent mean between girls and boys were *large* for emotional problems (*d* = 1.062) and prosocial behaviors (*d* = 0.622), and *small* (*d* = 0.116–0.280) for all others, according to the conventional criteria for interpretation [41]. In the model accounting for DIF, however, the effect sizes for the differences in latent means for emotional problems (*d* = 1.147) and conduct problems (*d* = 1.062) were *large*, and *small* to *medium* (*d* = 0.117–0.439) for all others. For conduct problems, the difference between the two models corresponds to a reduction of 0.833 in effect size, suggesting that disregarding DIF would lead to a substantial underestimation of the gender differences in conduct problems scores. Other changes in latent mean differences between the two models were small in magnitude and the pattern of gender differences in latent means was similar.

**Table 3:** Differences in latent factor mean between girls and boys when differential item functioning for gender is not accounted for, and when it is accounted for.

	Not accounting for differential item functioning		Accounting for differential item functioning	
	*Δ* Latent mean^a^	Cohen’s *d*	*Δ* Latent mean^a^	Cohen’s *d*
Emotional problems	-0.61**	1.062	-0.65**	1.147
Conduct problems	0.11**	0.280	0.43**	1.113
Hyperactivity/ inattention	-0.10**	0.188	-0.09**	0.181
Peer problems	0.07*	0.116	0.07*	0.117
Prosocial behaviors	-0.50**	0.622	-0.37**	0.439

^a^ For each subscale, the latent mean for girls is fixed to zero and used as reference. Negative values therefore indicate that boys have lower than girls on the latent SDQ scale, whereas positive values indicate that boys have higher scores than girls on the latent SDQ subscale.

* = *p* < .01

** = *p* < .001

We proceeded to test the model for structural equivalence by constraining the co-variance between the latent factors to be equal. Compared to the model in the previous step, the fit of the structural equivalence model was poorer (CFI = 0.918, TLI = 0.912, RMSEA = 0.057, CI [0.056, 0.058]) and the model fit was reduced more than the acceptable criteria of 0.002 thus suggesting structural non-invariance. Modification indices suggested that the co-variance constraint between the latent factors emotional problems and conduct problems should be freed, and this improved the fit of the model to an acceptable level (CFI = 0.919, TLI = 0.913, RMSEA = 0.057, CI [0.056, 0.058]) as the correlation between emotional problems and conduct problems was notably larger for boys (*r* = .488) than for girls (*r* = .310). See Table 4 for correlations between all the latent factors.

**Table 4:** Correlations between latent factors of the Strengths and Difficulties Questionnaire for girls (above diagonal) and boys (below diagonal).

	1	2	3	4	5
1. Emotional problems	–	.310	.525	.577	-.097
2. Conduct problems	.488	–	.799	.502	-.622
3. Hyperactivity/inattention	.534	.790	–	.245	-.341
4. Peer problems	.671	.568	.261	–	-.512
5. Prosocial behaviors	-.092	-.569	-.294	-.506	–

## Discussion

The present study examined the proposed factor structure of the SDQ in a sample of 16–18-year-old boys and girls, and tested whether the SDQ is measurement invariant across gender. The results suggest that a modified five-factor solution provides an acceptable fit to the data. Evidence of partial gender non-equivalence (i.e., measurement *non*-invariance) was found.

With regards to the factor structure of the SDQ, our results were in line with the findings from other studies supporting a five-factor model, one from Norway [9], and one from Spain [14] which have explored the factor structure by the use of CFA among older adolescents. Acceptable fit, however, was only obtained after accounting for one cross loading between the *tantrum* item and the emotional problems latent factor, and correlating the error terms between *fidgety* and *restless*, and between *distract* and *attends* in the latent hyperactivity-inattention factor. Most likely the detected correlated error terms represents minor factors (hyperactivity and inattention) within the general hyperactivity/inattention factor [13, 22] that leads to a poorer fit if they are not accounted for. Several studies have found a similar local dependency (correlated error terms) between especially fidget and restless within the hyperactivity/inattention factor [9, 11, 14, 22, 25]. Due to the consistency of this finding we propose that future studies of the SDQ includes these two local dependencies within the Hyperactivity/inattention factor to avoid misfit and improper factor loadings. Related to this, it is important to note that until we modified the model, we had a similar fit as Yao, Zhang, Zhu and Jing [23] who reported that the five-factor solution had a poor fit in this age group. It is tempting to speculate that their conclusions would have been more favourable if also they had taken these local dependencies within the hyperactivity/inattention factor into account.

In addition to the local dependencies we also located a cross loading consisting of a Conduct item–*tantrums* which also loaded on the Emotional factor in addition to the Conduct factor. As far as we know this cross loading is rather unique for the present study and probably represents a translation effect. Sanne, Torsheim and Heiervang [13] also located this cross loading in a study of the parent version of the SDQ, based on the first wave of the present longitudinal sample when the students were in grades 2–4. They point out that the item “… or hot tempers” in the CON1 item was translated “… eller dårlig humør,” which may be back-translated to “… or bad mood” ([13], p.354). In the current study, however, the updated Norwegian translation was used (which corresponds to the version available on the SDQ website). The item label “Often has temper tantrums or hot tempers” is in this version translated into “Jeg blir ofte sint og har kort lunte” which may also be back-translated into “I often get angry and have a short fuse”. Anger and temper outbursts may be behavioural manifestations of irritability [42] which is present in diagnostic criteria for both depression and anxiety disorders in the ICD-10 and DSM-IV, and has been found to be a strong predictor of depression and generalized anxiety disorder [43, 44]. It is thus not surprising that this item also loads on the Emotion factor and illustrates the challenges that may arise when translating questionnaires into other languages. Relatedly, several investigations have found measurement invariance in the SDQ across countries [16, 45, 46], highlighting how the responses to the self-report SDQ may be sensitive to cultural or geographical differences in language or meanings of the items in the SDQ.

As suggested by Stone et al. [18] the low internal consistency of the SDQ sub scales were acceptable (*α*s 0.71–82) as long as the ordinal nature of the data were taken into account. As shown in the present (*α*s 0.47–0.73) and in many other studies, the internal consistency, becomes deflated and poor for most of SDQ's sub scales if the items are treated as continuous through the use of Chronbach’s *α*.

The results did demonstrate partial measurement non-equivalence for gender; there was evidence of differential functioning for gender in eight of the items in the SDQ, and the correlation between emotional problems and conduct problems was higher for boys than girls. While most of the gender DIF effects were rather small and probably of little practical significance, three items had a noticeable lower threshold for girls compared to boys. Two of the items with noticeable DIF effects belonged to the Conduct factor and suggest that girls had a lower threshold of endorsing the items *tantrum* and *obeys* (reversely scored) than boys with a similar level of conduct problems. Given the fact that DIF effects are often caused by multidimensionality [47], the DIF effect of the *tantrum* item is at least partly caused by the fact that this item loaded on the Emotion factor in addition to the intended Conduct factor. As girls tend to have more emotional problems they will thus be more likely to endorse this particular item than a boy with a similar level of conduct problems as the item response is affected by both the conduct and the emotion factor. We are less certain as for why there was a particularly large gender DIF effect tied to the *obeys* item. But we suspect that it might reflect the possibility that the *obeys* item (reversed coded)—“I usually do as I am told” is not particularly well suited to assess conduct problems in this age group as its negation might assess healthy autonomy among 16–18-year-olds in addition to conduct problems. The final noticeable gender DIF effect related to *cares* and indicates that, conditioned on their level of prosocial behavior, girls will be more likely to endorse this item than boys. Interestingly, this gender DIF effect was also found by van de Looij-Jansen et al. [22] in a study of older adolescents in Netherland. A possible reason for this DIF effect is that this is the only prosocial item in the SDQ which does not only focus on *behavior* but also feelings for others (“I try to be nice to other people. I care about their feelings”)—a focus which might be particularly high for females [48].

The finding of partial non-equivalence for gender in the SDQ self-report version is in contrast with one other study where this has been explicitly tested in adolescence [14] and partly in agreement with another [22]. These studies used the criterion of ΔCFI< 0.01 proposed by Chen et al. [28] and Cheung and Rensvold [29] to determine whether the change in model fit was significant. Ortuño-Sierra et al [14] obtained a ΔCFI of < .01 for both configural and strong invariance, but reported only CFI rounded to the second decimal, and their results can therefore not be re-evaluated according to the more stringent criterion of ΔCFI< 0.002 for determining measurement invariance that was proposed by Meade, Johnson and Braddy [27]. van de Looij-Jansen et al. [22] actually obtained a CFI value greater than their specified cut-off (ΔCFI< 0.01) for the model testing strong factorial invariance across gender, but located only one gender DIF effect (*cares*–see above).

When evaluating models and measurement according to conventional cut-offs, selecting appropriate cut-offs become crucial. Researchers who rely on different criteria for making judgments about their models, may reach opposite conclusions from identical results. Both the ΔCFI< 0.01 proposed by Chen [28] and Cheung and Rensvold [29], and the ΔCFI< 0.002 proposed by Meade, Johnson and Braddy [27] are based on simulation studies, and the practical consequences of their application may not be immediately apparent. The current study used the more stringent criterion of these two, and demonstrated practical consequences of gender non-equivalence in terms of differential item functioning, latent mean score differences with large effect sizes, and correlations between emotional problems and conduct problems that were notably higher boys compared to girls. Had a more lenient criterion for determining measurement invariance been used, no further analysis would have been undertaken and these differences would have gone unnoticed.

There are several considerable strengths of this study, such as the large sample size, the comprehensive testing for measurement invariance for gender that rarely has been done for the SDQ, and the demonstrations of the practical consequences of gender non-equivalence.

However, there are also several limitations to the present study. The construct validity would have been strengthened by validating the SDQ against mental health instruments with age-appropriate questionnaires, but this was beyond the scope of the present study. While one of the strengths of the SDQ is the opportunity of having multi-informant responses, the present study is limited to adolescent report. Whether parental and teacher reports show the same factor structure for this age group or not awaits further investigation. The analyses are based on a large population based sample, however adolescents in school are overrepresented and this might restrict generalizability. Separate models were ran excluding those 76 participants that was not attending school in order to assess the appropriateness for the items in the SDQ for this group of adolescents. The results of the analyses of the samples with and without those adolescents not attending school were substantively similar, suggesting that, in this sample, including a small number of adolescents not in school does not alter the main findings. High scores on the SDQ have previously been associated with nonresponse in investigations using a sample similar to the current [49], and although the samples are not directly comparable, it is possible that participants with lower symptom scores are overrepresented in the current study.

One recommendation that follows from this finding is that researchers who test for measurement invariance, at least in models of comparable complexity to the SDQ, should evaluate change in model fit according to the ΔCFI< 0.002 criterion proposed by Meade, Johnson and Braddy [27]. Reductions of model fit of this magnitude may have practical consequences in terms of non-equivalent functioning across group variables as the current study demonstrates.

The consequences of gender non-equivalence in the SDQ will depend on the intended use of the instrument. With regards to within-gender comparisons of mean SDQ scores or investigations of how the SDQ score is related to other variables, the findings from the current study may be of little concern. However, between-gender comparisons on mean scores may be seriously biased by measurement invariance, and apparent gender differences in observed scores may result from measurement bias and not reflect actual gender-related differences on the underlying trait. The results from the current study suggest that this bias may especially relate to the conduct problems subscale of which the gender differences were seriously deflated if the differential item functioning was not taken into account. When the gender DIF effects were taken into account boys had a mean conduct problems score that was 1.113 standard deviations units higher than girls. This mean gender difference decreased to 0.280 standard deviation units if the DIF effects were not taken into account. According to Cohen’s [41] criteria these differences amount to large and small effect sizes, respectively, and underline the importance of testing whether the scale is measurement invariant. Due to the large impact of the DIF effects on the Conduct factor we advise against the use of using a simple sum score to assess conduct problems when using the Norwegian version of the SDQ as the translation of the “tantrum” item may not be optimally suited for adolescents.

With regards to using the SDQ to screen for mental health problems, and thereby identifying individuals in need of follow-up, using the conduct problems scale in itself may be problematic when there is only partial invariance [50]. Similarly, relying only on the total SDQ problems scale, which is based on the sum of the subscales and therefore includes the biased conduct problems subscale, may also be problematic for the same reason. Future research should assess the impact of the non-invariance on screening when using the SDQ.

The findings from the current study were in support of the proposed five-factor structure of the SDQ, demonstrating the utility of the instrument also among older adolescents. Importantly, the analyses did show evidence of partial gender non-equivalence, which has implications for its use as a screening instrument. Studies of measurement invariance of the SDQ are few in general, and further investigations of invariance for important characteristics such as gender, age and ethnicity are called for.
